# Supplementary material for: Content-rich biological network constructed by mining PubMed abstracts
Source: BMC Bioinformatics. 2004 Oct 8;5:147. doi: 10.1186/1471-2105-5-147 (PMC528731; doi:10.1186/1471-2105-5-147)
Supplement: Additional File 2 — The original results of the above study (non-essential files are deleted to keep the file size under the limit set by BMC bioinformatics). [file 1471-2105-5-147-S2.bz2 › chilibotAdditionalFile2/dip20/360ID9195914E5748/html/IL8_IL8RB.html]

 


 **IL8** and **IL8RB** 
  
Found 192 abstracts in PubMed, retrieved 20.  
 

 What does Google say? 
 PDF only 
| .edu only 

---

**Interactive relationship** (e.g. stimulation, inhibition, etc)

**Neutral relationship**- IL 8  [ **IL8** ]  Induced Migratory Responses through CXCR1 and CXCR2  [ **IL8RB** ]  Association with Phosphorylation and Cellular Redistribution of Focal Adhesion Kinase.  Ref: 12627953 Biochemistry, 2003
- The migration activating concentrations of 50 ng mL GCP  [ **IL8** ]  2 and NAP  [ **IL8** ]  2 induced less potent migratory responses and FAK phosphorylation in CXCR2  [ **IL8RB** ]  expressing cells as compared with IL 8  [ **IL8** ] .  Ref: 12627953 Biochemistry, 2003
- Characterization of the molecular interactions of interleukin 8  [ **IL8** ]  CXCL8  [ **IL8** ] , growth related oncogen alpha CXCL1 and a non peptide antagonist SB 225002 with the human CXCR2  [ **IL8RB** ] .  Ref: 12628493 Biochem Pharmacol, 2003
- CXCR1 and CXCR2  [ **IL8RB** ]  mediate migratory activities in response to IL 8  [ **IL8** ]  and other ELR CXC chemokines e.g., GCP  [ **IL8** ]  2 and NAP  [ **IL8** ]  2.  Ref: 12627953 Biochemistry, 2003
- IL 8  [ **IL8** ]  or CXCL8  [ **IL8** ]  activates the receptors CXCR1 IL 8RA and CXCR2  [ **IL8RB** ]  IL 8RB to induce chemotaxis in leukocytes, but only CXCR1 mediates cytotoxic and cross regulatory signals.  Ref: 12626541 J Immunol, 2003
- The CXCR2  [ **IL8RB** ]  is a GPCR that is activated, amongst the others, by the chemokines CXCL8  [ **IL8** ]  IL 8  [ **IL8** ]  and CXCL2 GROb to induce cell chemotaxis.  Ref: 12888558 J Biol ChemJ Biol Chem, 2003

**Non-interactive relationship** (e.g. studied together, co-existance, homology, etc.)

- We examined the expression of IL 8  [ **IL8** ]  and its two receptors CXCR1 and CXCR2  [ **IL8RB** ]  in 40 surgically resected human pancreatic cancer tissues and in three different human pancreatic cancer cell lines PANC 1, MIAPaCa 2 and Capan 2.  Ref: 12632066 Int J Oncol, 2003
- Thus in COPD, in addition to CXCL8  [ **IL8** ]  and CXCR1, CXCL5 and CXCR2  [ **IL8RB** ]  appear to play important roles in the airway neutrophilia characteristic of severe exacerbations.  Ref: 12857718 Am J Respir Crit Care MedAm J Respir Crit Care Med, 2003
- However, the contribution of polymorphisms of the IL 8  [ **IL8** ]  gene and its receptor genes CXCR 1 and CXCR 2  [ **IL8RB** ]  to human TB susceptibility remains untested.  Ref: 12870115 J Infect Dis, 2003
- In this study, we addressed the question of whether expression of the interleukin 8  [ **IL8** ]  receptors IL 8RA CXCR1 and IL 8RB CXCR2  [ **IL8RB** ]  is upregulated in H. pylori infected human gastric biopsy samples.  Ref: 12761119 Infect Immun, 2003
- The stimulation of CXCR1 and CXCR2  [ **IL8RB** ]  by IL 8  [ **IL8** ]  concentrations that result in migratory activation induced focal adhesion kinase FAK phosphorylation in a G alpha i dependent manner.  Ref: 12627953 Biochemistry, 2003
- Our data suggest that IL 8  [ **IL8** ]  directly enhanced endothelial cell proliferation, survival, and MMP expression in CXCR1 and CXCR2  [ **IL8RB** ]  expressing endothelial cells and regulated angiogenesis.  Ref: 12626597 J Immunol, 2003
- Finally, the mechanism of binding of a non peptide antagonist on CXCR2  [ **IL8RB** ]  was investigated it occurred through epitopes a which were disseminated within the receptor, b which differed according to the use of CXCL8  [ **IL8** ]  or CXCL1 as a competitor and c which did not necessarily overlap with agonist binding sites.  Ref: 12628493 Biochem Pharmacol, 2003
- Both IL 8  [ **IL8** ]  receptors, CXCR1 and CXCR2  [ **IL8RB** ] , are expressed extensively in the fetal intestine.  Ref: 12633567 Cytokine, 2002
- The mutants generated showed that a for CXCL8  [ **IL8** ]  binding, the N terminus of CXCR1 and the second extra cellular loop of CXCR2  [ **IL8RB** ]  are determinant, the N terminus of CXCR2  [ **IL8RB** ]  is not sufficient and the transmembrane domain seven is probably involved.  Ref: 12628493 Biochem Pharmacol, 2003
- The immunohistochemical analysis using specific antibodies demonstrated that positive staining for IL 8  [ **IL8** ] , CXCR1 and CXCR2  [ **IL8RB** ]  in surgically resected human pancreatic cancer was 5 5 and 65%, respectively.  Ref: 12632066 Int J Oncol, 2003
- In the present study, we examined the expression of chemokine receptors CXCR1 and CXCR2  [ **IL8RB** ]  receptors for interleukin 8  [ **IL8** ]  and CCR5 receptor for RANTES regulated on activation, normal T cell expressed and secreted, macrophage inflammatory protein MIP 1alpha, and MIP 1beta in human endometrium.  Ref: 12606476 Biol Reprod, 2003
- It is well known that chemokines and chemokine receptors, such as interleukin 8  [ **IL8** ]  and its receptors CXCR1 and CXCR2  [ **IL8RB** ] , play important roles in the pathogenesis of psoriasis.  Ref: 12823305 Clin Exp Dermatol, 2003
- By comparison with the NSC and S COPD groups, neutrophilia and gene expression for epithelial derived neutrophil attractant 78 CXCL5, interleukin 8  [ **IL8** ]  CXCL8  [ **IL8** ] , CXCR1 and CXCR2  [ **IL8RB** ]  were each up regulated in the E COPD group p&lt.  Ref: 12857718 Am J Respir Crit Care MedAm J Respir Crit Care Med, 2003
- Expression of the chemokine receptors CCR2, specific for MCP 1, and CXCR1 and CXCR2  [ **IL8RB** ] , specific for IL 8  [ **IL8** ] , were not altered by HCMV infection.  Ref: 12714640 Invest Ophthalmol Vis Sci, 2003
- In contrast, in CXCR2  [ **IL8RB** ]  RBL cells the migration attenuating concentrations of IL 8  [ **IL8** ]  induced promoted levels of FAK phosphorylation and different patterns of FAK phosphorylation on its six potential tyrosine phosphorylation sites, as compared to activating concentrations of the chemokine.  Ref: 12627953 Biochemistry, 2003
